# Reduced Prefrontal Gyrification in Carriers of the Dopamine D4 Receptor 7-Repeat Allele With Attention Deficit/Hyperactivity Disorder: A Preliminary Report

**DOI:** 10.3389/fpsyt.2019.00235

**Published:** 2019-04-25

**Authors:** Lena Palaniyappan, Martin J. Batty, Peter F. Liddle, Elizabeth B. Liddle, Madeleine J. Groom, Chris Hollis, Gaia Scerif

**Affiliations:** ^1^Robarts Research Institute, Western University, London, ON, Canada; ^2^Norfolk and Norwich University Hospital, Norwich, United Kingdom; ^3^University of Nottingham, Nottingham, United Kingdom; ^4^University of Oxford, Oxford, United Kingdom

**Keywords:** cortical thickness, genetics, inferior frontal gyrus, morphology, attention deficit/hyperactivity disorder

## Abstract

**Objective:** Structural and functional abnormalities have been noted in the prefrontal cortex of individuals with neurodevelopmental disorders such as attention deficit/hyperactivity disorder (ADHD). Cortical thickness and gyrification, both of which have been reported as abnormal in the prefrontal cortex in ADHD, are thought to be modulated by genetic influences during neural development. This study aimed to investigate the effects of a polymorphism of the dopamine DRD4 gene (the 7-repeat (7R) “risk” allele) on thickness and gyrification as distinct parameters of prefrontal cortical structure in children with ADHD.

**Method:** Structural images and genetic samples were obtained from 49 children aged 9–15 years (25 with ADHD and 24 matched controls), and measures of cortical thickness and gyrification for inferior, middle, and superior frontal cortex were calculated.

**Results:** A significant interaction between diagnosis and genotype on prefrontal gyrification was observed, largely driven by reduced inferior frontal gyrification in patients who carried the DRD4 7R allele. Furthermore, inferior frontal gyrification—but not thickness—related to everyday executive functioning in 7R allele carriers across groups.

**Conclusions:** Prefrontal gyrification is reduced in children with ADHD who also carry the DRD4 7R allele, and it relates to critical functional skills in the executive domain in carriers of the risk allele. More broadly, these effects highlight the importance of considering precise neurodevelopmental mechanisms through which risk alleles influence cortical neurogenesis and migration.

## Introduction

Attention deficit/hyperactivity disorder (ADHD) is a heterogeneous neuropsychiatric disorder characterized by developmentally inappropriate symptoms of inattention, impulsivity, and hyperactivity. The disorder is thought to affect around 1–5% of children and is highly heritable, with multiple genes of small effect contributing to the clinical phenotype (1). Although a number of genome-wide association studies (GWAS) [e.g., Refs. (2, 3)] have failed to show genome-wide significance in ADHD, a recent GWAS meta-analysis found associations with ADHD for 12 independent genome-wide significant loci (4). The dopamine D4 receptor (DRD4) gene, expressed primarily in limbic regions and the prefrontal cortex (PFC) (5), contains a 48-base-pair variable number tandem repeat in exon 3. A 7-repeat (7R) allele has been identified as a prominent risk allele for ADHD (6–10) and produces a blunted response to dopamine (11). At a neurochemical level, an abnormality in dopaminergic neurotransmission is well established in ADHD (12, 13) in keeping with the therapeutic effects of methylphenidate (MPH), an indirect dopamine agonist widely used to treat the disorder. ADHD has a strong neurodevelopmental basis, with numerous local and global brain abnormalities being observed (14) particularly in the cortical thickness of the prefrontal regions (15, 16). However, despite the convergence of evidence from genetic and pharmacological studies implicating the dopaminergic system, the relationship between genetic risk factors, dopaminergic imbalances, and the neurodevelopmental abnormalities observed in ADHD is most likely complex and remains unclear, but can be informed by models of genetic influences on cortical development (17, 18).

A bulk of the genetic polymorphisms identified to be associated with ADHD in genome-wide studies is functionally related to neural development (19). Animal studies have demonstrated a relationship between dopaminergic imbalance and alterations in cortical neurogenesis (20, 21). Dopamine plays a crucial role in the cell cycle of neural progenitors (22) as well as prefrontal cortex (PFC) functioning (23) and executive control (24), with the latter two heavily implicated in the pathophysiology of ADHD. A series of investigations seeking to identify the genetic control of cortical development suggest that subtle disturbances in the control of neurogenesis and migration can result in abnormalities affecting behavior that become apparent only during later postnatal life (25). Disturbances in early neurogenesis and neuronal migration can affect the process of cortical folding, resulting in abnormalities in cortical gyrification (26). Aberrations in cortical gyrification have been reported in initial investigations in children with ADHD (27, 28) and in longitudinal studies (29) though some studies have failed to observe such aberrations (30). The normal developmental trajectory of cortical gyrification is thought to be influenced by genetic mechanisms that are distinct from those controlling cortical volume or thickness (31, 32). Thus, cortical gyrification, and specifically its characteristics in PFC, is likely to be a potential intermediate phenotype reflecting aberrant neurodevelopment in ADHD and one whose mediation by dopaminergic imbalances and its genetic modifiers has not thus far been investigated.

A large genetic imaging study seeking to identify the mediating effect of neuroanatomical deficits in the relationship between the DRD4 7R risk allele and ADHD phenotype revealed lateral prefrontal thinning in patients with ADHD (33). The observed pattern of cortical thinning was comparable to differences noted in individuals carrying the 7R risk allele who did not have ADHD (33), suggesting that in the presence of risk alleles, the morphological abnormalities noted in the PFC of children with ADHD may be inflated. In a smaller study, reduced lateral prefrontal volume was also observed in patients who carry the risk allele (7). Cortical gyrification was not investigated in the above studies. Based on these observations (7, 33), we hypothesized that the 7R risk allele would be associated with alterations in the cortical thickness and gyrification of the PFC seen in ADHD. To this end, we investigated the effect of the DRD4 7R allele carrier status on both thickness and gyrification of the lateral PFC in children with ADHD and healthy controls.

## Materials and Methods

### Participants

Participants were recruited as part of an functional magnetic resonance imaging(fMRI)/electroencephalography (EEG) study reported elsewhere (34, 35) and described in detail in Batty et al. (16). Briefly, right-handed participants with a *Diagnostic and Statistical Manual of Mental Disorders, Fourth Edition* (*DSM-IV*) clinical diagnosis of ADHD (combined subtype) and an established positive response to methlyphenidate (MPH) were recruited from child psychiatry and community pediatric clinics in Nottinghamshire and Lincolnshire. Controls were recruited from primary and secondary schools in the Nottinghamshire region and matched for age ( ±6 months), sex, and socioeconomic status. Exclusion criteria included presence or history of head injury, psychosis, epilepsy, Tourette syndrome, autistic spectrum disorder, moderate or severe learning difficulties, or current use of non-stimulant or other psychotrophic medication (except melatonin). After a complete description of the study, written informed consent and verbal assent were obtained from parents and children, respectively. The study was approved by local Regional Ethics Committee and the Research and Development Departments of Nottinghamshire Healthcare NHS Trust and Lincolnshire Partnership NHS Foundation Trust.

Neuroimaging data from 54 children were available. A quality control inspection assessed images for gross structural abnormalities, motion, and other artifacts. Data from five subjects (all controls) were not included because of motion artifacts. In the final sample, we report data from 49 subjects with good quality neuroimaging and genotyping data; 25 subjects with ADHD [age (mean ± SD), 12.66 ± 1.78 years] and 24 healthy controls (12.93 ± 1.62 years).

### Neuropsychological Measures

Neuropsychological measures including IQ and reading ability were obtained from all participants as part of a testing battery (see **Table 1**). All parents completed the Behavior Rating Inventory of Executive Functioning (BRIEF) (36), a questionnaire of everyday behaviors associated with executive functioning. The BRIEF comprises 86 questions (e.g., “written work is poorly organised”), forming eight subscales (inhibit, shift, emotional control, initiate, working memory, plan/organize, organization of materials, monitor), which can be summed to generate an overall index of executive functioning, the Global Executive Composite (GEC) (36). Population-normed *T* scores (population mean = 50, SD = 10) are used to interpret the child’s executive functioning, with high scores indicating potential problems.

**Table 1:** Demographic characteristics of the sample.

	Group				*p* value (df)
	ADHD *n* = 25	(SD)	Control *n* = 24	(SD)	
Gender	M = 24		M = 23		0.75 (1)
Age (years)	12.66	(1.78)	12.93	(1.62)	0.45 (47)
Weight (kg)	43.96	(15.50)	51.17	(13.64)	0.09 (47)
Medication duration (months)	37.28	(23.71)	n/a		n/a
Methylphenidate dosage (mg/kg/day)	1.07	(.43)	n/a		n/a
FSIQ	91.36	(11.05)	104.79	(14.48)	**0.001** (47)
Digit span scaled	8.12	(3.35)	9.58	(3.34)	0.13 (47)
TOWRE: total score	88.96	(21.94)	98.33	(14.96)	0.088 (47)
Conners’ parent DSM total score	82.24	(7.52)	43.83	(3.37)	** <0.001** (47)
BRIEF (parent)-GEC	76.32	(8.08)	42.79	(7.55)	** <0.001** (47)
					
SES classification (*n*):					0.81 (3)
Higher professional	1		1		
Lower professional	5		5		
Self-employed	1		0		
Manual/unemployed	18		18		
Comorbid diagnoses:					
ODD	12		0		n/a
CD	6		0		n/a
GAD	4		0		n/a
Specific phobia	4		0		n/a
Reading	1		0		n/a
DCD	1		0		n/a
DRD4 7R carriers	11		7		
DRD4 7R non-carriers	14		17		0.377 (1)

FSIQ, full scale intelligence quotient; TOWRE, Test of Word Reading Efficiency; BRIEF-GEC, Behavior Rating Inventory of Executive Functioning–Global Executive Composite; SES, socioeconomic status; ODD, oppositional defiant disorder; CD, conduct disorder; GAD, generalized anxiety disorder; DCD, developmental coordination disorder; df, degree of freedom.

Some participants had more than one comorbidity.

### Magnetic Resonance Imaging Protocol

T1-weighted (T1W) brain images in the sagittal plane were obtained with a Philips Achieva 1.5-T MRI scanner with an eight-channel SENSE head coil using a 3D turbo field echo (TFE) sequence with the following parameters: 160 contiguous slices; TR/TE, 9.9/3.7 ms; matrix size, 256 × 256; voxel size, 1 × 1 × 1 mm. Head movement was minimized by the use of foam pads placed within the head coil.

### Surface Extraction

Surface extraction and cortical parcellation were carried out using FreeSurfer version 5.0 (37). The preprocessing was carried out according to the description available at http://surfer.nmr.mgh.harvard.edu/. Briefly, following skull-stripping and intensity correction, the gray–white matter (GM/WM) boundary for each cortical hemisphere was determined using tissue intensity and neighborhood constraints. The resulting surface boundary was tessellated to generate multiple vertices across the whole brain before inflating. Using a deformable surface algorithm guided by the gray–cerebrospinal fluid (CSF) intensity gradient, the resulting gray–white interface was expanded to create the pial surface. The inflated surface was then morphed into a sphere, followed by registration to an average spherical surface for optimal sulcogyral alignment. The neuroanatomical parcellations were obtained using the Destrieux sulcogyral based atlas, which follows the anatomical conventions of Duvernoy (38). Further description of this automatic extraction is available at http://surfer.nmr.mgh.harvard.edu/fswiki/DestrieuxAtlasChanges. The surface area of the right and left hemispheres was computed separately from the outer gray matter surface (pial surface) and summed up to obtain the total cortical surface area. Cortical thickness was measured using the method described by Fischl and Dale (39).

### Computing Gyrification

**Figure 1:**
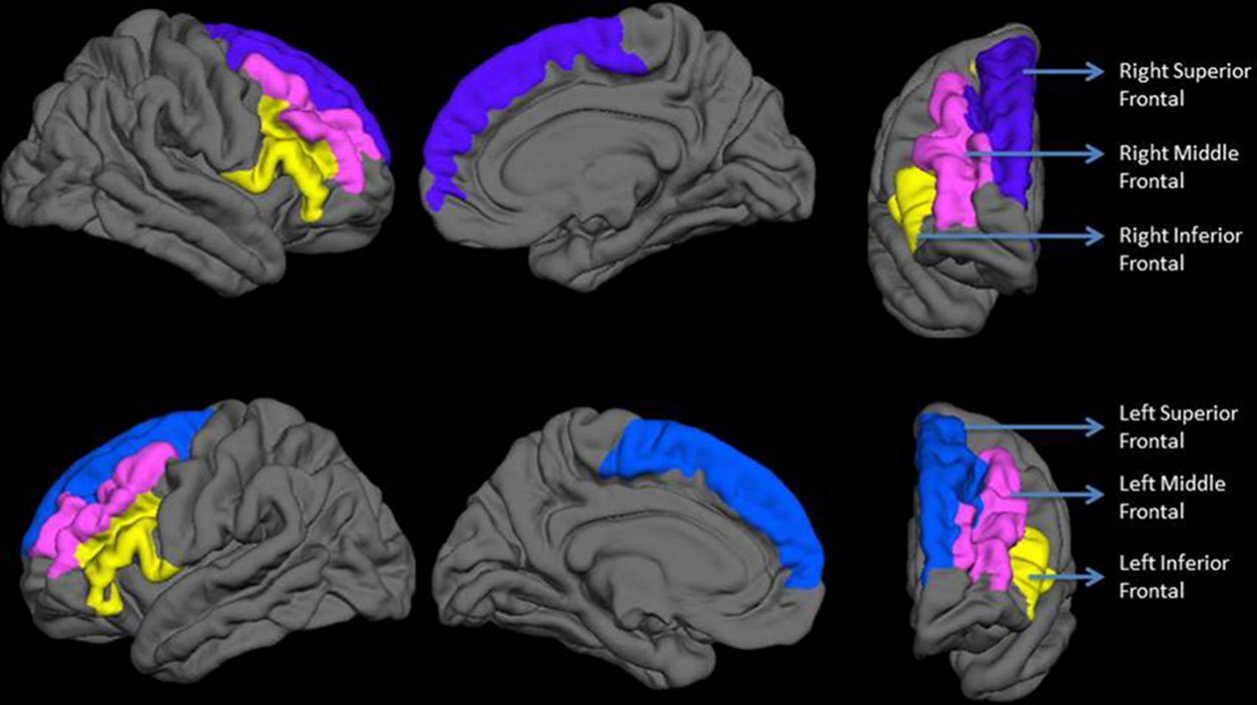
Regions included in the analysis. The top row shows lateral, medial, and anterior surfaces of the right hemisphere. The bottom row shows lateral, medial, and anterior surfaces of the left hemisphere. Labels are displayed on an average reconstructed FreeSurfer pial surface.

Vertex-wise quantification of cortical gyrification was computed using Schaer’s Local Gyrification Indices (LGIs) (40) using images reconstructed through the FreeSurfer pipeline. This approach has been previously employed to quantify gyrification abnormalities in localized regions of interest (41, 42). Schaer’s method is a vertex-wise extension of Zilles’ gyrification index, which gives a ratio of the inner folded contour to the outer perimeter of the cortex (43). The measure of LGI for each vertex on the pial surface reflects the amount of cortex buried in its locality (a value of 1 would indicate a flat pial surface). Mean LGI for anatomically meaningful sulcogyral regions can be obtained using the automated parcellation procedure from the FreeSurfer. For each anatomical region, the LGI value reflects a composite measure of the curvature, depth, and frequency of sulcal dips in the region (40). Given our hypotheses, we computed the LGI of the three lateral prefrontal sulcogyral divisions—superior frontal (composed of superior frontal sulcus and gyrus), middle frontal (composed of middle frontal sulcus and gyrus), and inferior frontal (including pars orbitalis, pars opercularis, and pars triangularis of inferior frontal gyrus along with the inferior frontal sulcus) as shown in **Figure 1**.

### Genetic Methods

Samples for genotyping were collected from participants using an Isohelix buccal swab (Web Scientific) or a mouthwash solution (10 µl 0.9% saline filter sterilized by 0.2 µl filter; Acrodisc Syringe filters) depending on subject preference. Samples were always collected following an abstinence from food and drink of at least 1 h.

DNA was extracted by centrifugation; 3,000 rpm for 10 min at 4°C. Pellets were resuspended in 500 µl of 10 mM NaCl/10 mM ethylenediaminetetraacetic acid (EDTA) and vortex mixed prior to centrifugation at full speed for 1 min. Pellets were resuspended in 500 µl of 50 mM NaOH, vortex mixed, and incubated at 100°C for 10 min. After centrifugation, 100 ml of 1 M Tris (pH 7.5) was added and vortex mixed. A 1:5 dilution of this stock was made and used in the PCRs.

The number of 48-bp repeats in each participant was determined using PCR across the repeat region. Primers and PCR conditions were as described by Nanko et al. (44), except that the sense primer was labeled with CY5. PCR products were electrophoresed on a Beckman CEQ8000 Genetic Analyzer, and the number of repeats was determined by size.

### Statistical Analysis

All statistical analyses were conducted using SPSS v.25 (IBM Inc). Demographic and clinical characteristics were compared using two-tailed *t* tests for continuous variables or chi-square (χ^2^) tests for categorical variables between patients and controls (CTRL) as well as between DRD4 7R carriers and non-carriers. A significance level of *p* < 0.05 was used for all tests.

Prefrontal gyrification was analyzed using a repeated-measures analysis of covariance (ANCOVA) with HEMISPHERE (left, right) and REGION (superior frontal, middle frontal, inferior frontal) as within-subject factors, DIAGNOSIS (ADHD vs. CTRL) and GENOTYPE [7R carriers or non-carriers, in line with Monuteaux et al. (7)] as between-subject factors, and total brain surface area (global covariate), age, and full scale intelligence quotient (FSIQ) as nuisance covariates. LGI covaries with total brain surface area (45) as well as IQ (46). At a population level, IQ (47) and total surface area are shown to be different between children with ADHD and those without ADHD (48, 49). To adjust for the possible confound of total surface area on prefrontal LGI, we used area as a covariate in line with prior studies (30, 50, 51). We assessed the main effects of DIAGNOSIS and GENOTYPE as well as the interaction effects in the model. A similar repeated-measures ANCOVA was used to investigate prefrontal thickness separately, with global cortical thickness as the global covariate and age and FSIQ as nuisance covariates. For this model, we included AGE as a covariate, in line with the recommendation by (52). In addition, we used global thickness as a covariate as global thickness scales with regional thickness values (53) and global thickness has been shown to be reduced in ADHD (15, 33). This approach is in line with prior MRI-based thickness studies that compare patients and controls (29, Palaniyappan et al., 2012). As the two global covariates (total surface area and global thickness) had a significant effect on the respective models, to ensure that our primary results (DIAGNOSIS × GENOTYPE) do not hinge on the inclusion of global covariates, we repeated the analyses after excluding the global covariates from the models. For all repeated-measures ANCOVA models, where the assumption of normality of residuals and/or homogeneity of variance was violated, *p* values for any follow-up tests were calculated from bootstrapped 95% confidence intervals for the parameter estimates (5,000 samples; bias-corrected and accelerated). Where the assumption of sphericity was violated, degrees of freedom were adjusted using the Huynh–Feldt correction.

*Post hoc* ANCOVAs were performed in carriers and non-carriers separately where a diagnosis × genotype interaction was found. Furthermore, percentage changes were calculated for each of the three frontal regions separately to identify the magnitude of group differences. Statistical power for ANCOVA was estimated with G*Power 3.1.3 (54). For main effects of DIAGNOSIS, GENOTYPE, and their interaction, our power to detect a large effect was 75.7%. We were therefore only adequately powered to detect large effect sizes, consistent with the effect sizes reported by Monuteaux (7) and Shaw (33), but we nonetheless highlight caution in interpreting null effects.

To understand the functional implications of the frontal gyrification in carriers and non-carriers, we performed correlations of the LGI (mean from both hemispheres for each region) to the BRIEF GEC score in carriers and non-carriers of the 7R allele. Given that there was no Diagnosis × Region interaction (see the section Results), correlations were performed in the entire sample not just in ADHD subjects. To reduce the probability of Type I error, a corrected *p* value of.008 (.05/6) was used.

## Results

*Participant Characteristics:* There were no significant difference in age, gender, or parental socioeconomic status (SES) between children with ADHD and controls (see **Table 1**).

*Genotyping:* There were no significant differences in prevalence of the DRD4 7R allele between children with ADHD and controls (*p* = 0.377; see **Table 1**).

The results of the analysis of covariance (ANCOVA) for cortical thickness and gyrification measures are shown in **Table 2**.

**Table 2:** Influence of attention deficit/hyperactivity disorder (ADHD) and DRD4 gene on the lateral prefrontal gyrification and thickness in ADHD.

Variable	Thickness			Gyrification		
	*F*	df	*p*	*F*	df	*p*
Hemisphere	0.94	1,42	0.34	0.001	1,42	0.97
Diagnosis	5.52	1,42	**0.02**	0.19	1,42	0.57
Genotype	0.19	1,42	0.67	0.18	1,42	0.73
Global covariate	93.24	1,42	**<0.001**	16.37	1,42	**<0.001**
Diagnosis × Genotype	0.01	1,42	0.93	5.447	1,42	**0.024**
Diagnosis × Hemisphere	0.60	1,42	0.45	0.337	1,42	0.56
Diagnosis × Regions	0.88	2,84	0.42	0.299	2,84	0.43
Diagnosis × Genotype × Regions	0.66	1,42	0.52	6.017	2,84	**0.004**
FSIQ	0.26	1,42	0.62	0.001	1,42	0.97
Age	0.26	1,42	0.61	0.20	1,42	0.66

*Cortical Thickness:* For prefrontal thickness, there was a significant main effect of DIAGNOSIS, whereby the ADHD group had a thinner PFC compared to healthy controls; *F*(1,42) = 5.52, *p* = 0.023 (controls: mean [SD] = 2.836 [0.064] mm; ADHD: mean [SD] = 2.794 [0.055] mm). The DIAGNOSIS × GENOTYPE interaction was not significant (*F* = 0.007; *p* = 0.934). Removing the global covariate did not alter the results [DIAGNOSIS: *F*(1,43) = 8.4, *p* = 0.006; DIAGNOSIS × GENOTYPE: *F*(1,43) = 0.31, *p* = 0.58; see **Table 3**]. Assumptions of homogeneity and normality of the residuals were not violated for these models.

**Table 3:** Effect of diagnosis on lateral prefrontal thickness in carriers and non-carriers of DRD4 7R allele.

Region	DRD4 Carriers					7R Non-carriers				
	Controls		Patients			Controls		Patients		
	Mean LGI	(SE)	Mean LGI	(SE)	% change	Mean LGI	(SE)	Mean LGI	(SE)	% change
Superior frontal	2.911	(0.035)	2.830	(0.028)	−2.8%	2.915	(0.024)	2.874	(0.026)	−1.4%
Middle frontal	2.640	(0.027)	2.596	(0.021)	−1.7%	2.655	(0.019)	2.582	(0.020)	−2.7%
Inferior frontal	2.948	(0.034)	2.939	(0.27)	−0.3%	2.956	(0.023)	2.928	(0.025)	−0.9%

*Gyrification:* A significant DIAGNOSIS × GENOTYPE interaction was observed for the gyrification index; *F*(1,42) = 5.45, *p* = 0.024. This interaction indicated that, while in non-7R carriers, mean LGI was slightly lower in the control group than in the ADHD group, in the 7R carriers, mean LGI was lower in the ADHD group. Removing the global covariate did not alter the ANCOVA results [DIAGNOSIS: *F*(1,43) = 1.37, *p* = 0.25; DIAGNOSIS × GENOTYPE: *F*(1,43) = 3.15, *p* = 0.036]. There was also a significant DIAGNOSIS × GENOTYPE × REGION effect, *F*(1.2,48.8) = 6.017, *p* = 0.014.

**Figure 2:**
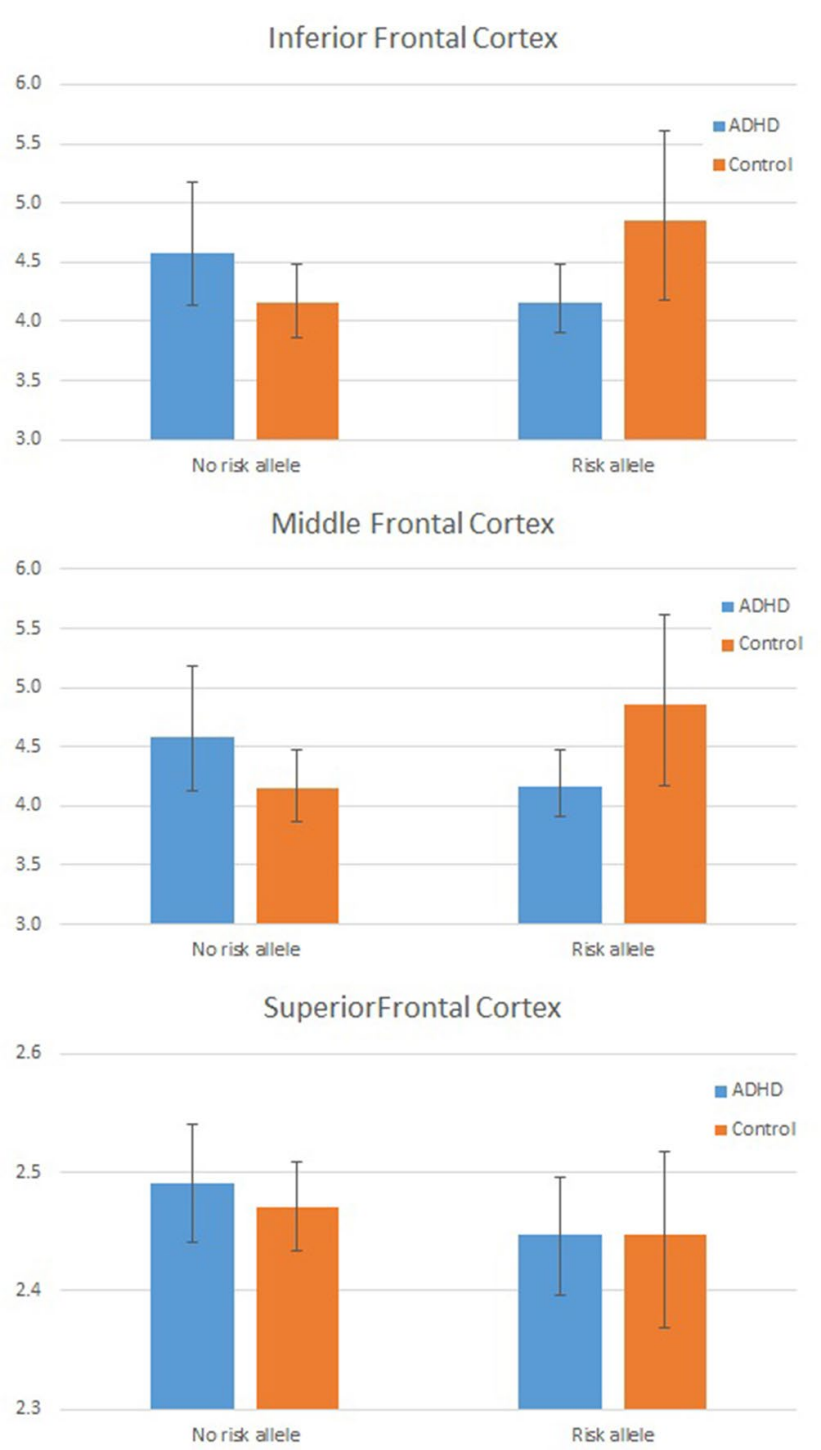
Plot showing individual Local Gyrification Index (LGI) values by Group and Genotype: In frontal cortex overall (left panel) and for inferior frontal cortex (right panel). LGI values are adjusted for age, full scale intelligence quotient (FSIQ), and total pial area [covariates used in the analysis of covariance (ANCOVA)].

As the assumption of homogeneity of variance and normality of residuals was violated in these models, we tested these interactions further using a series of bootstrapped univariate factorial ANCOVAs, first with global mean frontal LGI as the dependent variable, and then, to test the three-way interaction, with mean LGI in each frontal region (inferior, middle, and superior frontal cortex) in turn as the dependent variable. For the DIAGNOSIS × GENOTYPE interaction, the effect remained significant (*p* = 0.042 for interaction parameter). Results from each region in turn indicated that this interaction was largely driven by a significant interaction effect in inferior frontal cortex (*p* = 0.019 for the interaction parameter). These data are shown graphically in **Figure 2** and **Table 4**.

**Table 4:** Effect of diagnosis on lateral prefrontal gyrification in carriers and non-carriers of DRD4 7R allele.

Region	DRD4 Carriers					7R Non-carriers				
	Controls		Patients			Controls		Patients		
	Mean LGI	(SE)	Mean LGI	(SE)	% change	Mean LGI	(SE)	Mean LGI	(SE)	% change
Superior frontal	2.448	(0.033)	2.448	(0.026)	0%	2.471	(0.023)	2.491	(0.024)	+0.80%
Middle frontal	3.150	(0.049)	3.0442	(0.039)	−3.4%	3.101	(0.034)	3.120	(0.037)	+0.61%
Inferior frontal	4.853	(0.280)	4.160	(0.224)	−14.3%	4.153	(0.195)	4.581	(0.178)	+9.4%

LGI, Local Gyrification Index.

An analysis of simple main effects (with the global covariates) revealed that the observed effects on gyrification were driven by a significant difference in the risk carriers, with children with ADHD showing a significant reduction in prefrontal gyrification, *F*(1,15) = 5.031, *p* = 0.04 (mean [SD] CTRL = 3.497 [0.275]; ADHD = 3.196 [0.271]), with no significant effect for the global covariate, *F*(1,15) = 3.28, *p* = 0.09. There was a significant DIAGNOSIS × REGION effect, *F*(1.17,17.5) = 5.43, *p* = 0.027. As the variances for the inferior frontal LGI were not equal between the two groups, we used Kruskal–Wallis *H* test to confirm a significant effect of diagnosis on inferior frontal LGI (*H* = 5.8, *p* = 0.016) among carriers.

In non-carriers, there was no significant diagnostic effect, *F*(1,28) = 2.28, *p* = 0.10 (mean [SD] CTRL = 3.236 [0.292]; ADHD = 3.414 [0.292]), but a significant effect for the global covariate, *F*(1,28) = 14.1, *p* = 0.001. There was no significant DIAGNOSIS × REGION effect among non-carriers, *F*(1.09,30.6) = 2.29, *p* = 0.14. As the residuals for the inferior frontal region were not normally distributed, we used Kruskal–Wallis *H* test to confirm the lack of a significant effect of diagnosis on inferior frontal LGI (*H* = 0.76, *p* = 0.38) among non-carriers.

We also noted that there was no significant effect of genotype among both controls [*F*(1,21) = 4.1, *p* = 0.06] and children with ADHD [*F*(1,22) = 2.59, *p* = 0.12]. Kruskal–Wallis *H* test was not significant among both control subjects (*H* = 3.51, *p* = 0.06) and children with ADHD (*H* = 0.76, *p* = 0.38).

*Behavioral Correlates:* A significant negative correlation was observed among carriers of the risk allele (*r* = −0.67, df = 17, *p* = 0.002), whereby children with high scores on the BRIEF had a lower inferior frontal LGI. Importantly, this correlation was not observed among the non-carriers (*r* = 0.20, df = 30, *p* = 0.28) or in the other regions (superior/middle frontal) in either group. We used Fisher’s *r*-to-*Z* conversion followed by a *t* test to assess the difference in inferior frontal LGI-BRIEF correlation between the two groups. We noted a significant difference (*z* = −3.15, *p* = 0.0015), confirming the genotype-specific relationship between real-world executive functioning and inferior frontal gyrification.

## Discussion

Using structural MRI to investigate the interaction between genotype (DRD4) and brain structure in ADHD, we found that children with ADHD who are carriers of the 7R allele had a significant reduction in frontal gyrification compared to typically developing controls with the 7R allele. This reduction appears to be largely driven by a hypogyric inferior frontal gyrus in children with ADHD who carry the 7R “risk” allele. These results support our hypothesis that the DRD4 7R allele influences the structure of the PFC in the presence of ADHD. Interestingly, there was a trend towards hypergyric inferior frontal gyrus among the healthy 7R carriers compared to the healthy non-carriers. The diagnosis dependent effect of the risk allele on brain structure highlights the polygenetic influences on cortical neurogenesis and supports the “multiple genes of small effect” model of ADHD (55). This effect might also be mediated by the inclusion of only participants with ADHD combined subtype and the inclusion of oppositional defiant disorder and conduct disorder, present in three quarters of the ADHD sample, as studies have shown stronger associations with ADHD and DRD4 7R in combined ADHD (10) and ADHD with comorbid conduct problems (56).

As expected, and consistent with previous work (33), children with ADHD had thinner PFC than matched controls. Also consistent with this previous study, genotype did not affect cortical thickness differentially more in ADHD cases compared to controls. However, we observed an association between morphometric changes (gyrification) in the inferior frontal cortex and the DRD4 7R allele, and this was specific to ADHD cases. Another study investigating morphometric changes associated with DRD4 7R did not include inferior frontal cortex (7), but did however report a significant reduction in superior prefrontal volume in carriers of the risk allele among adults with ADHD.

For the first time, our results show that children with ADHD—but not controls—have reduced frontal gyrification in the presence of the 7R risk allele, and these differences emerge predominantly in inferior frontal areas. A prevailing notion is that the polymorphisms implicated by genome-wide association studies of ADHD contribute to cortical development (19). It is possible that DRD4 7R influences the early phase of corticogenesis, through a hitherto unknown mechanism, resulting in an aberrant neurodevelopmental trajectory in those who go on to develop ADHD. In fact, the anatomical effect of DRD4 7R on inferior frontal regions appear to be more pronounced in earlier than later stages of neurodevelopment, as shown by Schweren et al. (57) in a volumetric study. Though a causal relationship cannot be established from this cross-sectional study, it is worth noting that longitudinal structural neuroimaging studies investigating cortical thickness support the notion that ADHD is associated with delayed prefrontal maturation (15). Similarly, other longitudinal studies have also found reductions in cortical volume in frontal regions in children and young people with ADHD (29), primarily driven by reduced surface area rather than reduced cortical thickness. In both instances, these differences in frontal regions are likely to be driven by atypical neuronal proliferation at an early stage of development. A normal increase in inferior frontal cortical complexity is noted between ages 6 and 16 years (58), raising the possibility that the reduced gyrification observed in the risk allele carriers with ADHD might be related to a delay in cortical maturation, in keeping with models of maturational delay in ADHD (59).

We observed a significant diagnosis × genotype × region effect for gyrification, with patients who are risk carriers showing a pronounced reduction in the LGI of inferior frontal gyrus (IFG). This suggests that the dopaminergic influence on the development of IFG in ADHD may be distinct from those on other lateral frontal regions. The inferior frontal cortex is considered to play an important role in inhibitory control in both healthy individuals (60) and children with ADHD (61). Inferior frontal gray matter reduction, especially in the right hemisphere, is associated with behavioral deficits commonly observed in ADHD (62), while damage to right IFG has been associated with poor inhibitory control (63). In the present study, there was no effect of hemisphere on cortical thickness or gyrification. Given our power limitations, these may have been too subtle effects for us to detect. Furthermore, while some studies have emphasized the importance of right IFG in inhibitory control, evidence for lateralization of GM deficits in IFG in ADHD is equivocal (16, 64). There is some suggestion that the functional changes observed in this region could be specific to ADHD (61) and could be modified to some extent by pharmacological manipulations (65). Our observations suggest that, among carriers, reduced inferior frontal gyrification, but not the gyrification of other two frontal regions, is linked to poor real-world executive functioning measured using BRIEF. Lack of such correlation in non-carriers reaffirms our hypothesis that frontal gyrification may mediate the risk conferred by 7R allele. Notably, thickness did not relate to behavioral correlates such as BRIEF and other neuropsychological measures (16).

Although the current results are strengthened by the inclusion of a well-defined clinical sample including only patients with *DSM-IV* combined type ADHD [corresponding to International Classification of Diseases, Tenth Revision (ICD-10) hyperkinetic disorder] and well-matched controls, some limitations must nonetheless be acknowledged. While the sample size is similar to or larger than that of many other morphometry studies (7, 27, 28, 66), our design was only sufficiently powered to detect large-sized effects and was not optimized to explore the effects of other variables, such as the relationship between symptom severity and gyrification. Negative findings, such as the lack of genotype effect on gyrification and thickness should be interpreted cautiously and would benefit from further investigation in larger samples. In addition, investigating the extent to which gyrification is influenced solely by ADHD or comorbid disorders or an interaction of the two requires greater power. Moreover, while reductions in gyrification in children with ADHD have been demonstrated by some groups (28, 29), others [e.g., Ref. (30)] have found no group differences in local gyrification. While the inclusion of only combined type ADHD and an almost entirely male sample reduce heterogeneity, the extent to which the findings can be generalized to other ADHD subtypes and to girls with ADHD is unclear, particularly given the differential effects of DRD4 with ADHD subtype (10). Finally, patients with ADHD were entered onto the study on the basis of their current use of and established positive response to methylphenidate (MPH), an indirect dopamine agonist. The extent to which MPH might influence gyrification, particularly in those with the 7R “risk” allele would require the inclusion of a medication-naive sample. However, it is unlikely that the reduction in frontal gyrification would be mediated by MPH for two reasons. Firstly, gyrification begins during the early stages of intrauterine life, from around 16 weeks (67) and is largely completed during early postnatal development, prior to the onset of stimulant medication. Secondly, structural abnormalities in children taking medication for ADHD are typically reduced relative to non-medicated children with the disorder (68, 69). Thus, the structural aberrations observed in this sample of medicated patients are more likely to be an underestimate of the extent of the pathophysiology.

It is important to consider the lack of significant association between DRD4 variants and ADHD in the recent large genome-wide association study (4), though some of the other affected loci (FOXP2, DUSP6) may have a role in synaptic dopamine regulation. It is also likely that the DRD4 polymorphism interacts with other genetic determinants of brain development, thus influencing the brain structure in ADHD. Larger genome-wide examination of the relationship between ADHD-related common variants and gyrification is needed to address this issue.

In conclusion, our findings show for the first time that reduced prefrontal gyrification is an important variable when investigating the ADHD phenotype in the presence of the DRD4 7R risk allele. In carriers of the risk allele, it also relates to everyday functional skills that are severely affected in ADHD. Investigating in detail multiple parameters of cortical morphometry can potentially elucidate the complex findings in ADHD genetics by revealing how the cortical developmental trajectory is modulated by genetic risk factors in the presence of the disorder. Uncovering the interaction between dopaminergic polymorphisms and cortical neurogenesis and migration holds the promise of a fuller understanding of the converging etiological influences in ADHD.

## Ethics Statement

After a complete description of the study, written informed consent and verbal assent were obtained from parents and children, respectively. The study was approved by local Regional Ethics Committee and the Research and Development Departments of Nottinghamshire Healthcare NHS Trust and Lincolnshire Partnership NHS Foundation Trust.

## Author contributions

MB and LP wrote the first draft of the manuscript and performed the statistical analyses. LP carried out the FreeSurfer analysis. PL, MG, EL, CH, and GS contributed to the study design and provided intellectual input into the manuscript.

## Funding

This work was partly supported by the Opportunities fund from the Academic Medical Organization of Southwest Ontario (LP). This work was supported by a grant from the Wellcome Trust (grant number: 076448/Z/05/Z) to CH (principal investigator).

## Conflict of Interest Statement

LP reports personal fees from Janssen Canada, Otsuka Canada, SPMM Course Limited, UK, Canadian Psychiatric Association; book royalties from Oxford University Press; investigator-initiated educational grants from Janssen Canada, Otsuka Canada and Sunovion outside the submitted work.

The remaining authors declare that the research was conducted in the absence of any commercial or financial relationships that could be construed as a potential conflict of interest.
